# Supplementary material for: MetaRibo-Seq measures translation in microbiomes
Source: Nat Commun. 2020 Jun 29;11:3268. doi: 10.1038/s41467-020-17081-z (PMC7324362; doi:10.1038/s41467-020-17081-z)
Supplement: Supplementary file 10 — Supplementary Data 7 [file 41467_2020_17081_MOESM10_ESM.zip › File2/Confidence_VeryHigh_Taxonomy/113107_out.krona.html]

Javascript must be enabled to view this page.

members
magnitude
magnitudeUnassigned
count
unassigned
taxon
rank

113107\_out

9

9
2
superkingdom

9
phylum
976

8
200643
class

order
171549
8

family
171552
8


SRS017916\_contig\_number\_contig-100\_9588.9588
genus
1
838
8


SRS016541\_contig\_number\_10465
1
28135
species


SRS023914\_contig\_number\_5020
species
1947225
1

species
1262929
1

SRS104912\_contig\_number\_1452


SRS024663\_contig\_number\_10013SRS049896\_contig\_number\_2194SRS053356\_contig\_number\_46741SRS1041116\_contig\_number\_4656
363265
species
4

1
species
1953172

SRS049959\_contig\_number\_contig-100\_36006.124523
